# Concentrated Electrolytes Widen the Operating Temperature Range of Lithium‐Ion Batteries

**DOI:** 10.1002/advs.202101646

**Published:** 2021-07-23

**Authors:** Jianhui Wang, Qifeng Zheng, Mingming Fang, Seongjae Ko, Yuki Yamada, Atsuo Yamada

**Affiliations:** ^1^ Key Laboratory of 3D Micro/nano Fabrication and Characterization of Zhejiang Province School of Engineering Westlake University Hangzhou 310024 China; ^2^ Department of Chemical System Engineering University of Tokyo Tokyo 113‐8656 Japan; ^3^ Elements Strategy Initiative for Catalysts and Batteries Kyoto University Kyoto 615‐8245 Japan; ^4^ School of Chemistry South China Normal University Guangzhou 510631 China

**Keywords:** concentrated electrolyte, lithium‐ion batteries, robust solid electrolyte interphase, wide temperature range

## Abstract

The operating temperatures of commercial lithium‐ion batteries (LIBs) are generally restricted to a narrow range of −20 to 55 °C because the electrolyte is composed of highly volatile and flammable organic solvents and thermally unstable salts. Herein, the use of concentrated electrolytes is proposed to widen the operating temperature to −20 to 100 °C. It is demonstrated that a 4.0 mol L^−1^ LiN(SO_2_F)_2_/dimethyl carbonate electrolyte enables the stable charge–discharge cycling of a graphite anode and a high‐capacity LiNi_0.6_Co_0.2_Mn_0.2_O_2_ cathode and the corresponding full cell in a wide temperature range from −20 to 100 °C owing to the highly thermal stable solvation structure of the concentrated electrolyte together with the robust and Li^+^‐conductive passivation interphase it offered that alleviate various challenges at high temperatures. This work demonstrates the potential for the development of safe LIBs without the need for bulky and heavy thermal management systems, thus significantly increasing the overall energy density.

## Graphical abstract

Owing to the highly stable solvation structure, concentrated electrolyte enables the stable operation of LiNi_0.6_Co_0.2_Mn_0.2_O_2_|graphite full cells in a wide temperature range from −20 to 100 °C, which alleviates various challenges faced by commercial dilute electrolytes. This study demonstrates the potential to build a safe battery system without the bulky and heavy thermal management system, thus significantly increasing the overall energy density.

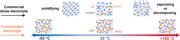

## 1 Introduction

**Figure 1:**
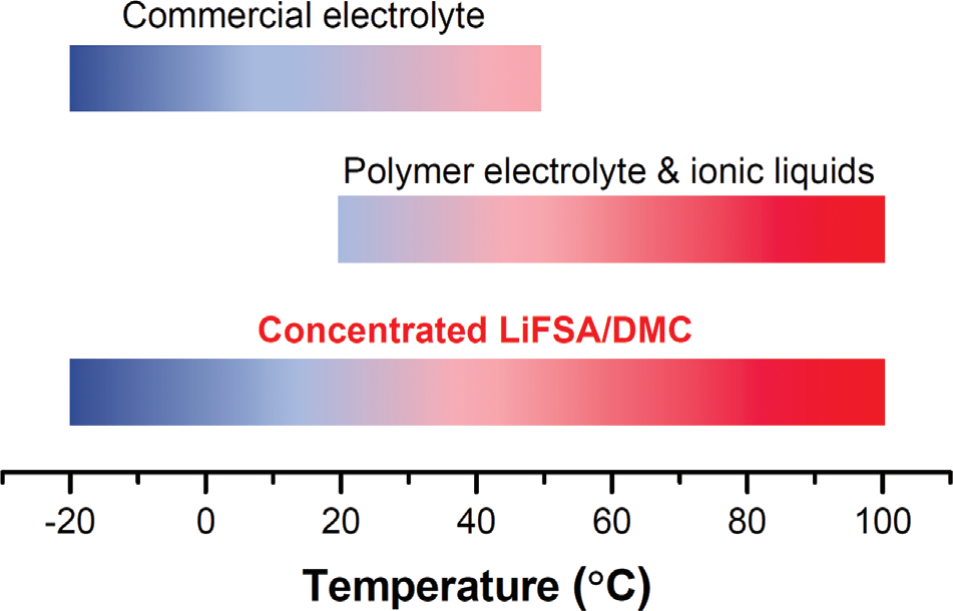
Operating temperature ranges of LIBs. Commercial 1 M LiPF_6_/ethylene carbonate:dimethyl carbonate (DMC) electrolyte can operate in a temperature range of −20 to 55 °C. Polymer electrolytes and ionic liquids have better rate and cycling performance at high temperatures of >60 °C, but their performance below room temperature is much poorer than that of the commercial electrolyte. The concentrated lithium bis(fluorosulfonyl)amide (LiFSA)/DMC electrolyte reported here exhibits both outstanding cycling performance at 100 °C and superior rate capability at −20 °C compared to the traditional electrolyte. Ceramic conductors are excluded from the comparison because they are still in an early stage of research, and there are several technical issues that must be overcome, for example, limited/unstable contact between electrode and electrolyte. In addition, there have been no benchmarked commercial products, which suggests that a practical electrolyte requires certain plasticity.

Lithium‐ion batteries (LIBs), initially commercialized in 1991, now dominate the power source market, covering portable consumer electronics to large‐scale electric vehicles and power‐grid systems. In recent years, LIB performance, including the energy density, rate capability, and cycling stability, have been improved considerably,^[^ ^1^, ^2^, ^3^ ^]^ but the operating temperature range remains narrow, that is, discharging between −20 and 55 °C and charging between 0 and 45 °C (**Figure** **1**).^[^ ^4^ ^]^ At high temperatures (>55 °C), the batteries suffer from rapid performance degradation and elevated risk of fire or even explosion (thermal runaway) because of the thermally susceptible organic electrolytes. Meanwhile, at low temperatures (<0 °C), they suffer from capacity loss and risk of internal short circuit caused by lithium metal plating on the graphite anode.^[^ ^5^, ^6^, ^7^ ^]^

The high sensitivity of LIB performance to temperature significantly limits large‐scale applications. For example, electric vehicle battery packs must be equipped with a battery thermal management system (BTMS) that enables the battery to operate within the safest and optimal temperature range (10 to 40 °C).^[^ ^8^, ^9^, ^10^ ^]^ The BTMS generally includes a cooler, heater, heat exchanger, sensors, pipelines, and pumps/fans, which take up additional space and weight, thus significantly reducing the energy density and increasing the cost of the battery system. Moreover, the BTMS has another key disadvantage: poor temperature measurement accuracy and poor thermal conductivity, which compromise the system response speed and properties.^[^ ^8^, ^10^ ^]^ When the environmental temperature reaches either >45 °C (e.g., in a hot summer) or <0 °C (e.g., in cold winter), the charging current must be reduced considerably to avoid potential safety incidents, resulting in much longer charging times. Despite these in‐built protections, safety‐related incidents involving electric vehicles repeatedly occur in hot and cold weather.

Improving the temperature tolerance of LIBs is an effective approach to enhancing battery stability, safety, and utilization efficiency compared to BTMS protection. Recently, remarkable progress has been made in expanding the low‐temperature limit of battery operation by introducing a self‐heating device^[^ ^11^ ^]^ or altering the electrolyte ingredients.^[^ ^12^, ^13^, ^14^, ^15^ ^]^ However, these efforts do not effectively improve the stability of high‐temperature operation, especially at >70 °C. In fact, for large‐scale applications of LIBs, elevating the upper‐temperature limit is highly desirable because significant heat is generated during battery operation, and this heat cannot dissipate rapidly inside the battery pack, resulting in a steady increase in temperature beyond the battery operating limit. If the battery itself can tolerate a high temperature, the requirement of BTMS to cool the battery system would be considerably reduced since the large temperature gradient between the battery and environment can effectively help self‐cooling.

However, the intrinsic drawbacks of commercial state‐of‐the‐art lithium‐ion electrolytes make the above target challenging.^[^ ^16^ ^]^ Lithium‐ion electrolytes typically comprise 1.0 M (mol L^−1^) LiPF_6_ salt dissolved in a mixed solvent of ethylene carbonate (EC) and linear carbonates. These organic carbonates are highly volatile and flammable, although LiPF_6_ also suffers from thermal instability at temperatures above 60 °C.^[^ ^17^ ^]^ Another critical issue arises because of the solid electrolyte interphase (SEI); this is a passivation film that forms on graphite anodes because of the decomposition of the EC solvent, and it plays an essential role in enabling the reversible lithiation/delithiation at low potentials (close to 0 V versus Li^+^/Li). Unfortunately, conventional SEIs are also thermally unstable, decomposing at 80 °C and leading to continuous reductive decomposition of the electrolyte with the generation of heat and gas products.^[^ ^18^ ^]^ Moreover, a series of physical and chemical chain reactions inside the battery can be triggered, such as an increase in the battery internal pressure, mechanical deformation of the battery configuration, and acceleration of heat propagation towards thermal runaway.^[^ ^19^, ^20^ ^]^ These factors cause the rapid degradation of the electrochemical performance (generally observed for graphite anodes at temperatures above 55 °C),^[^ ^21^, ^22^, ^23^ ^]^ as well as severe safety risks. Consequently, the high‐temperature operation of LIBs is strictly prohibited. To overcome this limitation, it is necessary to develop new electrolytes to replace the conventional electrolyte for LIBs.

Polymer electrolytes and ionic liquids have attracted a great deal of attention owing to their superior battery performance at elevated temperatures (i.e., 60–100 °C).^[^ ^24^, ^25^ ^]^ However, polymer electrolytes encounter high electrode–electrolyte interfacial resistance, and ionic liquids suffer from high viscosity and poor ionic conductivity.^[^ ^1^, ^26^, ^27^ ^]^ In addition, it remains a great challenge to preserve battery performance in subzero temperatures. In recent years, various attractive physicochemical and electrochemical properties have been demonstrated for concentrated electrolytes (usually >3 M), and these properties are dramatically distinct from those of conventional dilute electrolytes, for example, low flammability and volatility, fast charging/discharging rate, robust interfacial compatibility, and superior lithium dendrite suppression ability.^[^ ^28^, ^29^, ^30^, ^31^, ^32^, ^33^, ^34^, ^35^, ^36^, ^37^, ^38^ ^]^ These excellent features of concentrated electrolytes may support a much wider operating temperature, which is particularly desirable for large‐scale integrated application, while few studies have worked on this aspect thus far. Recently, a concentrated electrolyte was reported to enable a potable‐type LiCoO_2_|graphite battery to work at 90 °C,^[^ ^39^ ^]^ while the thermal stability issue of SEI on the graphite anode was not studied and the fundamental physicochemical understanding behind the electrolyte has not been explored yet in this paper.

In this work, we report a concentrated electrolyte that can significantly increase the operating temperature for LIBs. We selected a combination of thermally stable lithium bis(fluorosulfonyl)amide (LiFSA) and oxidatively stable dimethyl carbonate (DMC) as a model system because this mixture enables the reversible intercalation of Li^+^ ions into graphite anodes at both low and high concentrations, thus providing a useful comparative study. In our previous work, we reported that this concentrated LiFSA/DMC electrolyte realized a high‐voltage LIB by overcoming transition metal dissolution at high voltages.^[^ ^33^ ^]^ Herein, we demonstrate this concentrated electrolyte can also efficiently alleviate various challenges that are encountered by conventional dilute electrolytes at extreme temperatures: 1) insufficient stability of the electrolyte itself and the electrolyte‐electrode interphases at both high and low temperatures; 2) severe gas evolution and transition metal dissolution at high temperatures; 3) sluggish charge‐discharge kinetics at low temperatures. Consequently, this electrolyte enables the reversible charge–discharge of high‐energy‐density LiNi_0.6_Co_0.2_Mn_0.2_O_2_ (NCM622)|graphite full cells in a wide temperature range of −20 to 100 °C. Our finding shows that the use of a concentrated electrolyte can remarkably widen the operating temperature of LIBs from the current range of −20 to 55 °C to a new, wider range of −20 to 100 °C, demonstrating the potential to build highly efficient LIB systems without the need for BTMSs.

## 2 Results and Discussion

### 2.1 Temperature‐Dependent Physicochemical Properties

**Figure 2:**
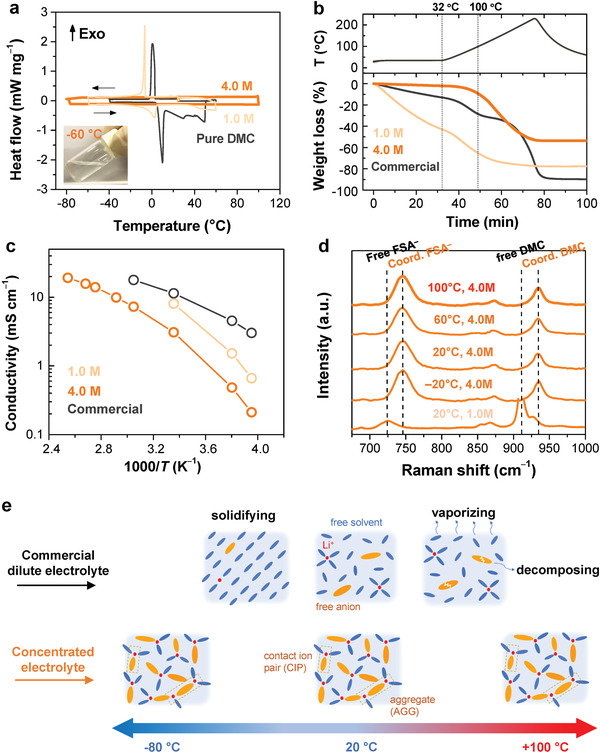
Physicochemical properties dependent on temperatures. a) DSC curves of pure DMC solvent, concentrated (4.0 M), and dilute (1.0 M) LiFSA/DMC electrolytes. Inset shows a digital image of the concentrated electrolyte after being stored at −60 °C for 2 h. b) Weight losses of concentrated (4.0 M) and dilute (1.0 M) LiFSA/DMC electrolytes and a commercial electrolyte (1.0 M LiPF_6_/EC:DMC (1:1 by vol.)) in an Ar flow upon heating. c) Ionic conductivities of different electrolytes at different temperatures. d) Raman spectra of concentrated (4.0 M) and dilute (1.0 M) LiFSA/DMC electrolytes at various temperatures. For the concentrated electrolyte, Raman spectra are collected at a temperature range from −20 to +100 °C while for the dilute electrolyte, Raman spectrum is only collected at 20 °C due to its narrow stable temperature window. e) Schematics of the solvation structures in commercial dilute electrolyte and concentrated electrolyte at a temperature range from −80 to 100 °C.

Concentrated (4.0 M) and dilute (1.0 M) electrolytes were prepared by dissolving LiFSA in DMC in an Ar‐filled glove box. The salt‐to‐solvent molar ratios were calculated to be 1:1.9 and 1:10.8 for 4.0 M and 1.0 M LiFSA/DMC solutions, respectively. Differential scanning calorimetry (DSC) was used to evaluate the thermal behavior of various solutions. As shown in **Figure** **2a**, pure DMC and 1.0 M LiFSA/DMC show sharp exothermic peaks at 4 and −7 °C during cooling, respectively, which were assigned to the crystallization or solidification of the liquid samples. Instead, no exothermic or endothermic peak was observed for the 4.0 M LiFSA/DMC electrolyte, suggesting it can maintain fully liquid state within the measuring temperature range from −80 to 100 °C, which was attributed to the absence of free solvent in the concentrated electrolyte that makes it behave like ionic liquid. Moreover, we also examined the thermal stability of this solution by thermogravimetry (TG). As shown in Figure 2b, the weight loss of 4.0 M LiFSA/DMC upon heating to 100 °C was approximately 8%, which is much lower than those of the 1.0 M LiFSA/DMC (i.e., 70%) and commercial 1.0 M LiPF_6_/EC:DMC (i.e., 30%). Furthermore, the latter two 1.0 M solutions even showed large weight losses of 45% and 13%, respectively, at room temperature in 20 min because of the high volatility of DMC. On this basis, the thermal stability is considerably improved for the concentrated electrolyte, which is beneficial for the high‐temperature operation of LIBs. In addition, the ionic conductivity of 4.0 M LiFSA/DMC is 3.1 mS cm^−1^ at 25 °C, increasing to 19 mS cm^−1^ at 120 °C (shown in Figure 2c and **Table** **1**). For the dilute electrolytes, we could not measure the true ionic conductivities over 70 °C because of their high volatility. One can imagine that a safety incident would occur if a battery is employed with these highly volatile electrolytes at such high temperatures. At −20 °C, the conductivity of 4.0 M LiFSA/DMC is 0.21 mS cm^−1^, which is an order of magnitude lower than that of the commercial electrolyte.

**Table 1:** Ionic conductivities of 1.0 M and 4.0 M LiFSA/DMC and commercial 1.0 M LiPF_6_/EC:DMC (1:1 by vol.) at different temperatures

	Ionic conductivity [mS cm^−1^]							
Electrolyte	−20 °C	−10 °C	25 °C	55 °C	70 °C	90 °C	100 °C	120 °C
Commercial	3.0	4.5	11	18	20*	‐	‐	‐
1.0 M	0.66	1.5	8.1	11*	‐	‐	‐	‐
4.0 M	0.21	0.48	3.1	7.3	9.9	14	16	19

* These values were underestimated because a significant amount of electrolyte was ejected from the cell by the accumulated pressure inside the cell at elevated temperatures.

It is generally accepted that physicochemical properties of solution are determined by its solution structure. The remarkable stability of concentrated 4.0 M LiFSA/DMC solution as observed in the wide temperature range from −80 to +100 °C should be ascribed to its peculiar three‐dimensional network structure that is completely different from conventional dilute solution that is dominated by free‐state solvent molecules. As shown in Figure 2d, the dilute solution of 1.0 M LiFSA/DMC is mainly composed of free DMC molecules and free FSA^−^ anions, whereas, the concentrated solution of 4.0 M LiFSA/DMC is composed of coordinated DMC and FSA with almost absence of free‐state species, which is consistent with the previous results.^[^ ^33^ ^]^ However, whether such a unique solvation structure for the concentrated solution can be maintained at cold temperature or extremely high temperature remains puzzling to the community. Particularly, at high temperatures, the coordinated moieties of contact ion pairs and aggregates would be possibly altered and thus destroy the associated network structure. To figure it out, we performed Raman spectroscopy measurements of the 4.0 M LiFSA/DMC solution at temperatures up to 100 °C. Clearly, both Raman bands of DMC and FSA^−^ maintain unchanged within the measuring temperature range from −20 to 100 °C, indicating the peculiar solution structure of the concentrated solution keeps stable even at extreme temperatures. Based on these thorough discussions, the solvation structures of both concentrated 4.0 M LiFSA/DMC and 1.0 M commercial electrolytes at different temperature were schematically summarized in Figure 2e. For 1.0 M commercial electrolyte with free‐state solvents and anions as the dominant component, the crystallization will occur when temperature goes below −20 °C, moreover, the electrolyte will vaporize as well as be decomposed when temperature rises above 80 °C. Therefore, a battery employed with a 1.0 M commercial electrolyte can only be operated within a narrow temperature range. For concentrated 4.0 M LiFSA/DMC electrolyte with little free‐state solvent and anion, the fully liquid state as well as their solvation structure were perfectly maintained from −80 to 100 °C, enabling the operation of battery in a wide‐temperature range.

### 2.2 Wide‐Temperature Operation of LIB

**Figure 3:**
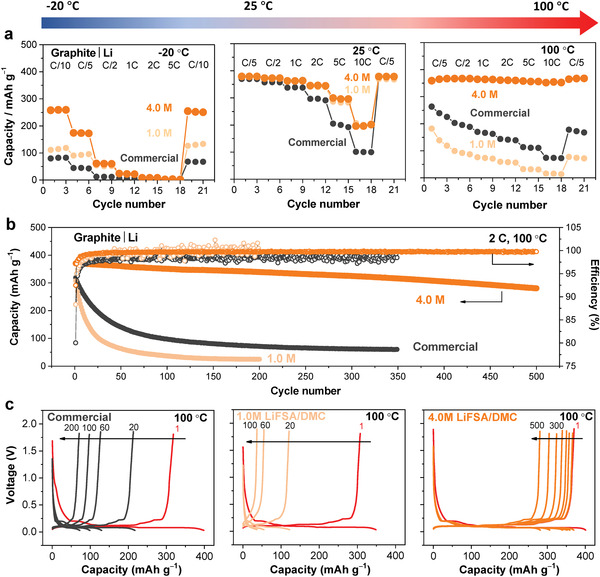
Wide‐temperature operation of a graphite electrode. a) Rate performance of graphite | Li half‐cells using lab‐made concentrated (4.0 M) and dilute (1.0 M) LiFSA/DMC electrolytes and a commercial electrolyte (1.0 M LiPF_6_/EC:DMC (1:1)) at different temperatures. b) Cycling performance of the half‐cells at a high temperature of 100 °C at 2 C rate. c) Selected charge–discharge curves for the half‐cells at 100 °C and 2 C rate. The 1 C‐rate corresponds to 372 mA g^−1^ on the weight basis of the graphite electrode. Charge and discharge are operated at the same temperature including the operation at −20 °C, which is different from other works on low‐temperature operations that are discharged at low temperatures but charged at room temperature.

Graphite is the most widely used anode material in commercial LIBs. As mentioned above, its electrochemical performance is profoundly affected by the SEI film on the surface, but this film is extremely sensitive to temperature. Hence, we first examined its temperature‐dependent performance. **Figure** **3a** shows the rate performance of graphite|Li half‐cells with various electrolytes at −20, 25, and 100 °C. At 25 °C, all three electrolytes yielded a reversible capacity of approximately 350 mAh g^−1^ at ≤1 C rate. In contrast, at higher rates (>2 C), the commercial electrolyte showed a lower capacity than those of 4.0 and 1.0 M LiFSA/DMC mixtures. However, the ionic conductivity of the commercial electrolyte was one order of magnitude higher than that of the concentrated electrolyte (3.0 versus 0.21 mS cm^−1^), indicating the rate performance is not simply dominated by the ionic conductivity. Lithium intercalation into a graphite electrode involves not only the transport of Li^+^ ions in the bulk electrolyte but also the desolvation of Li^+^ on the SEI and the subsequent transport of desolvated Li^+^ ions through the SEI layer.^[^ ^40^, ^41^ ^]^ The electrochemical impedance spectra of the graphite electrodes show that 4.0 M LiFSA/DMC resulted in a large electrolyte bulk resistance (*R*_el_) because of its low ionic conductivity. However, it could be fully compensated by much smaller SEI resistance (*R*_SEI_) and charge‐transfer resistance (*R*_ct_), resulting in a comparable or even better rate capability than 1.0 M LiFSA/DMC or the commercial electrolyte at 25 °C (Figure S1 and Table S1, Supporting Information).

Cooling to −20 °C dramatically increased all contributions to the resistance, and the cells with both 4.0 and 1.0 M LiFSA/DMC exhibited a much lower *R*_SEI_ than that with the commercial electrolyte (Table S1, Supporting Information). In addition, 4.0 M LiFSA/DMC resulted in a lower *R*_ct_ than 1.0 M LiFSA/DMC. Consequently, 4.0 M LiFSA/DMC led to the best rate capability for the graphite electrode, whereas the commercial electrolyte showed the worst rate capability (see Figure 3a). Overall, the better rate capability of 4.0 M LiFSA/DMC than 1.0 M LiFSA/DMC is mainly due to rapid Li^+^‐ion desolvation arising from their different Li^+^‐ion coordination environments^[^ ^33^ ^]^ or high Li^+^‐ion activity, whereas the better rate capability of both LiFSA/DMC electrolytes compared to the commercial electrolyte could be attributed to rapid Li^+^‐ion transport in the SEI, which is most likely associated with the different SEI compositions (discussed later).

Elevating the temperature theoretically accelerates Li^+^‐ion transport and, thus, enhances the rate performance. However, when tested at 100 °C, the two dilute electrolytes (1.0 M LiFSA/DMC and the commercial electrolyte) caused lower capacities of the graphite electrodes than at 25 °C. Furthermore, their capacities declined quickly in just a few cycles, which is consistent with the literature results,^[^ ^21^, ^22^, ^23^ ^]^ suggesting that these dilute electrolytes do not allow the operation of a graphite electrode at high temperatures. In contrast, 4.0 M LiFSA/DMC offered excellent rate performance for the graphite electrode (360 mAh g^−1^ at 10 C rate), demonstrating its suitability for battery operation in a much wider temperature range with significantly enhanced kinetics.

To demonstrate the superior performance of 4.0 M LiFSA/DMC at high temperatures, long‐term charge–discharge cycling tests were carried out at 100 °C. As shown in Figure 3b,c, the graphite|Li half‐cell with 4.0 M LiFSA/DMC exhibited capacity retention of 76% with an average Coulombic efficiency of approximately 99.7% over 500 cycles, thus outperforming the cells with 1.0 M LiFSA/DMC and the commercial electrolyte remarkably.

**Figure 4:**
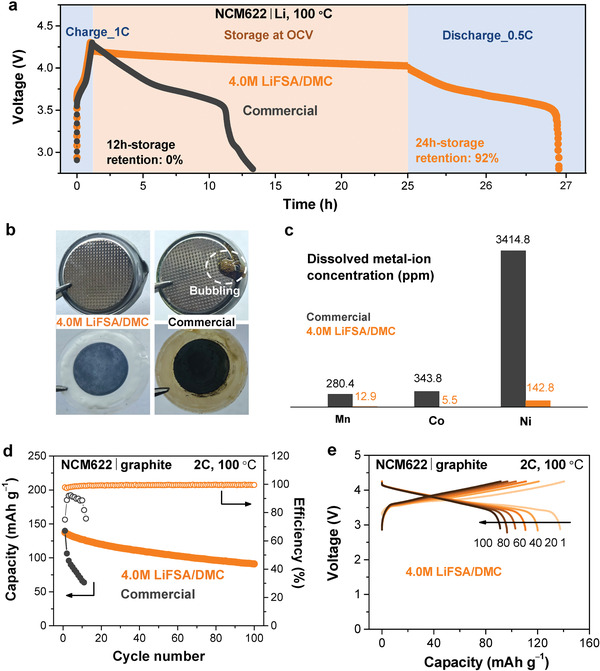
High‐temperature operation of an NCM622 electrode in half‐cell and full cell. a) High‐temperature storage test for NCM622|Li cells using 4.0 M LiFSA/DMC and a commercial electrolyte (1.0 M LiPF_6_/EC:DMC (1:1)). b) The digital images of the NCM622|Li coin cells after a cycling test at 100 °C. When crack the coin cell using pliers in the glovebox, considerable brown‐color liquid is immediately bubbling out from the cell with the commercial electrolyte, evidencing a large amount of gas had been produced that dramatically increased the internal pressure of the cell. And the separator turned to brown color, implying that the commercial electrolyte had undergone a serious decomposition at this high temperature. In sharp contrast, the cell parts with the concentrated 4.0 M LiFSA/DMC electrolyte remained very clear. c) The dissolved metal‐ion concentration in the electrolytes of the NCM622|Li coin cells after a cycling test at 100 °C. d) Cycling performance of the NCM622|graphite full cells using 4.0 M LiFSA/DMC and the commercial electrolyte at 100 °C and 2 C. e) Selected charge–discharge voltage curves for the full cell with 4.0 M LiFSA/DMC at 100 °C and 2 C. The 1 C‐rate corresponds to 160 mA g^−1^ on weight basis with respect to the NCM622 cathode.

Furthermore, we carried out a high‐temperature storage test to check if this electrolyte is compatible with the NCM622 cathode material at high voltages. The NCM622|Li cells using commercial electrolyte and 4.0 M LiFSA/DMC electrolyte were first charged to 4.3 V at 1 C and then kept at 100 °C at open‐circuit voltage. As shown in **Figure** **4a**, the cell using commercial electrolyte underwent severe self‐discharge with no capacity retained after 12 h. While for the cell using 4.0 M LiFSA/DMC electrolyte, no obvious self‐discharge was observed with a 92% capacity retention being retained, suggesting its excellent capability of retaining capacity when being stored at high temperatures.

Then, we performed a cycling test for NCM622|Li half‐cell at 100 °C. A reasonable capacity retention of 83% was achieved over 100 cycles for the cell using the 4.0 M LiFSA/DMC electrolyte (see Figure S2, Supporting Information), indicating the potential application of this concentrated electrolyte at high voltages. After that, the cycled cells were disassembled in the glovebox to check the changes that occurred inside the cells. As demonstrated in Figure 4b, when we cracked the coin cell using pliers, considerable brown‐color liquid was immediately bubbling from the cell using 1.0 M commercial electrolyte, indicating severe gas evolution by the thermal decomposition of electrolytes, which may lead to explosion when the pressure exceeds cell limit. Furthermore, it can be seen that the cell components (e.g., electrolyte, electrode, and separator) turn to brown color (Figure 4b), which was ascribed to the thermal instability of LiPF_6_ and severe parasitic reactions between electrodes and electrolyte at high temperature. In sharp contrast, the cell parts maintained clear without bubble or any obvious change in 4.0 M LiFSA/DMC electrolyte, suggesting excellent stability itself as well as toward other cell components at 100 °C, which would effectively reduce the thermal runaway risk. In addition, to evaluate the dissolved metal‐ion concentration, the electrolytes of the cycled cells were examined by an inductively coupled plasma mass spectrometer (ICPMS). The results (Figure 4c) indicate that the concentrations of Mn, Co, and Ni in the concentrated electrolyte were only 1/60 to 1/20 as those in the commercial electrolyte, clearly evidencing the concentrated electrolyte can effectively suppress the transition metal dissolution of the cathode at high temperature, which is also beneficial for high‐temperature operation of the battery.

Having confirmed the stable operation of both NCM622|Li and graphite|Li half‐cells at 100 °C, 4.0 M LiFSA/DMC was further applied to an NCM622|graphite full cell. As shown in Figure 4d, the full cell demonstrated capacity retention of 66% over 100 cycles with an average Coulombic efficiency of approximately 99.5% at 100 °C, which is much better than that obtained using the commercial electrolyte. Even though there was a slight increase in polarization during the charging process (Figure 4e), the electrochemical performance indicates great potential for LIB operation at approximately 100 °C using the concentrated electrolyte. Besides, the concentrated electrolyte also demonstrated a stable charge‐discharge operation of the full cell at a low temperature of −20 °C, superior to the commercial electrolyte (see Figure S3, Supporting Information).

### 2.3 Robust Electrolyte–Electrode Interphase

**Figure 5:**
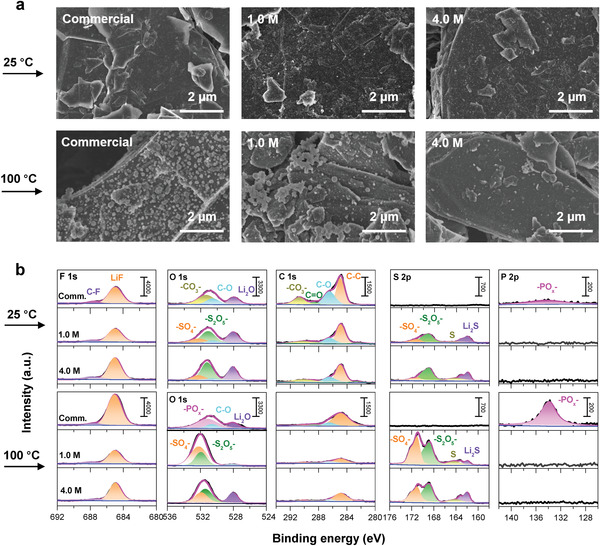
Surface analysis of the graphite electrodes after cycling in various electrolytes and temperatures. a) SEM images of the graphite electrodes after cycling at 25 and 100 °C. b) X‐ray photoelectron spectra of the graphite electrodes cycled at 25 and 100 °C. The electrolytes studied here include concentrated (4.0 M) and dilute (1.0 M) LiFSA/DMC electrolytes and a commercial electrolyte (1.0 M LiPF_6_/EC:DMC (1:1)). Profound changes of the morphology and composition can be found on the graphite electrodes cycled in both dilute electrolytes of 1.0 M LiFSA/DMC and commercial 1.0 M LiPF_6_/EC:DMC (1:1), whereas little change can be found on the electrode cycled in the concentrated electrolyte, demonstrating that the concentrated electrolyte enables robust interphase at high temperatures.

To obtain a deeper understanding of the function of the concentrated electrolyte, scanning electron microscopy (SEM) and X‐ray photoelectron spectroscopy (XPS) were used to analyze the surface of the graphite electrodes after cycling at 25 and 100 °C. As shown in **Figure** **5a**, no significant differences were found in the SEM images for all graphite electrodes that had been cycled at 25 °C. However, after cycling in the two dilute electrolytes at 100 °C, the graphite surfaces were covered with many particles having diameters of 100–300 nm. In contrast, for the electrode cycled in 4.0 M LiFSA/DMC, the surface morphology showed little change after cycling at 100 °C. Together with the superior electrochemical performance offered by concentrated electrolytes at high temperature (Figure 3), we can make the conclusion that the SEI produced in the concentrated electrolyte is more robust than that produced in dilute electrolytes (commercial or 1.0 M LiFSA/DMC).

In addition to the surface morphology, the chemical compositions of the electrode surfaces were obtained by analysis of the XPS spectra, as shown in Figure 5b. For the cell operated at 25 °C, the SEI generated from the commercial electrolyte was mainly composed of LiF, Li_2_CO_3_, and LiOR (organic compounds), which is consistent with the literature,^[^ ^16^, ^42^, ^43^ ^]^ whereas the SEIs generated from 4.0 M and 1.0 M LiFSA/DMC contained a significant amount of sulfur compounds that were derived from FSA^−^ anions. As previously reported,^[^ ^36^ ^]^ these sulfur compounds may have a higher Li^+^ conductivity than that of LiF, Li_2_CO_3_, and LiOR, which is supported by the much lower *R*_SEI_ observed for the graphite electrodes in the LiFSA/DMC electrolytes (Figure S1, Supporting Information). Moreover, Figure 5b shows that concentrated 4.0 M LiFSA/DMC led to a higher content of sulfur compounds and LiF. Obviously, the chemical compositions of the SEIs on these three graphite electrodes differed. However, the passivated graphite electrodes could be cycled well at 25 °C, indicating that the SEI components are stable at room temperature.

After charge–discharge operation at 100 °C, the chemical compositions of the electrode surfaces underwent remarkable changes in the two dilute electrolytes. As shown in Figure 5b, for the electrode with the commercial electrolyte, the F1s and P2p peaks increased in intensity considerably, whereas the intensities of the C1s peaks decreased, indicating that the surface products generated at 100 °C were mainly derived from LiPF_6_ rather than EC. This finding is different from that at room temperature, wherein EC instead of LiPF_6_ is the primary SEI‐forming agent. These unusual phenomena can be attributed to two factors: 1) the EC‐derived organic components generally decompose at >80 °C, and, thus, EC alone cannot produce a stable SEI at high temperatures, and 2) LiPF_6_ is also thermally unstable and suffers from severe decomposition at 100 °C. Consequently, this leads to thick and rough interphase between the graphite electrode and the electrolyte (see the electrode morphology in Figure 5a), which then induces large polarization and a fast capacity decay during both rate and cycling tests (See Figure 3).

In the case of 1.0 M LiFSA/DMC, the SEI generated at 25 °C was derived from both LiFSA and DMC. Similarly, the solvent‐derived organic components are thermally unstable; thus, the surface products at high temperatures should be derived mainly from LiFSA. This assumption is supported by the data in Figure 5b: the intensities of the peaks in the F1s, O1s, and S2p spectra increase significantly for the graphite electrode after operation at 100 °C. However, by comparing the spectra of S2p and O1s after cycling at 100 and 25 °C, the surface components at 100 °C were different from those at 25 °C, suggesting an alternative passivation reaction at the elevated temperatures. One reason could be from the poor thermal stability of the dilute electrolyte (see Figure 2a); elevating the temperature may induce the thermal degradation of the electrolyte or some side reactions between the electrolyte and electrode that produce different components on the graphite surface. According to the SEM images shown in Figure 5a, these components generated at 100 °C tend to form particles rather than a compact film, which is not sufficient to passivate the graphite electrode and, thus, results in poor cycling performance, as also observed with the commercial electrolyte.

Unlike those in the dilute electrolytes, the chemical composition and surface morphology of the graphite electrodes remained almost the same after cycling at 25 and 100 °C in the concentrated 4.0 M LiFSA/DMC electrolyte, demonstrating that the electrode–electrolyte interphase is robust and stable. These outstanding properties obtained in the concentrated electrolyte could be caused by the following three effects: 1) the salt‐derived SEI is thermally more stable owing to its lower content of unstable organic components, 2) the reduction products of the concentrated electrolyte show little change in both composition and morphology in a wide temperature range, which facilitates in situ healing of the SEI at any temperature, and 3) the concentrated electrolyte itself has a much better thermal stability, which further stabilizes the electrode–electrolyte interphase at high temperatures.

**Figure 6:**
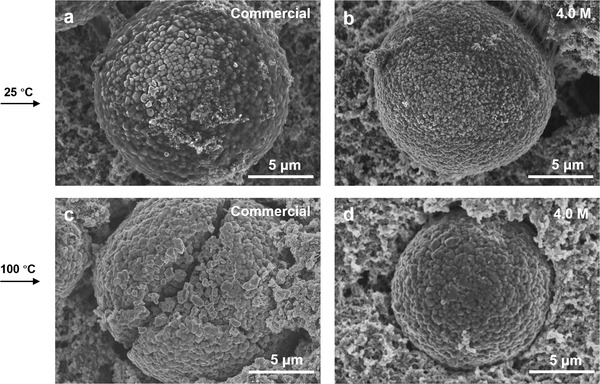
SEM images of the NCM622 electrodes after cycling in various electrolytes and temperatures. a,b) are SEM images of NCM622 electrodes cycled at 25 °C in a commercial electrolyte (1.0 M LiPF_6_/EC:DMC (1:1)) and concentrated electrolyte (4.0 M LiFSA/DMC), respectively. c,d) are SEM images of NCM622 electrodes cycled at 100 °C in the commercial electrolyte and the concentrated electrolyte, respectively.

Meanwhile, the morphologies of NCM622 electrodes after cycling at 25 and 100 °C were investigated by SEM. As shown in **Figure** **6a**,b and Figure S6, Supporting Information, the microstructures of NCM622 electrodes after cycling at 25 °C in both commercial and 4.0 M LiFSA/DMC electrolytes were well‐maintained. While for cycling at 100 °C, the NCM622 electrode using commercial electrolyte experiences severe fracture (Figure 6c and Figure S7, Supporting Information), which is likely due to the thermal instability of LiPF_6_ that results in the generation of corrosive HF as well as the subsequent induced gas evolution on electrode/electrolyte interface, causing the structural damage of cathode. In contrast, for the electrode using 4.0 M LiFSA/DMC, the microstructure showed little change after cycling at 100 °C (Figure 6d and Figure S7, Supporting Information), indicating the concentrated electrolyte can effectively prevent the structural degradation of cathode, which may be attributed to the very stable solvation structure of concentrated electrolyte that can restrain the parasitic reactions between electrode and electrolyte.

## 3 Conclusions

We have demonstrated that a concentrated 4.0 M LiFSA/DMC electrolyte enables the stable operation of an NCM622|graphite LIB in a wide temperature range from −20 to 100 °C, thus outperforming the conventional dilute electrolyte. This outstanding performance is attributed to the multiple advantages offered by concentrated electrolytes, including the highly stable solvation structure of the electrolyte itself in a wide temperature range and highly Li^+^‐ion‐conductive and robust electrolyte–electrode interphases. Specifically, at a low temperature of −20 °C, the highly Li^+^‐conductive LiFSA‐derived SEI generated by the concentrated electrolyte can compensate for its relatively poor bulk ionic conductivity, thus leading to the fast and stable cycling of the battery. At a high temperature of 100 °C, the high thermal stability of both electrolyte and electrolyte–electrode interphases effectively reduces the gas evolution and transition metal dissolutions, and leads to an improved electrochemical performance that far exceeds that of the conventional dilute electrolyte. The remarkably increased upper‐temperature limit from 55 to 100 °C effectively enhances the thermal stability and safety of the battery system because when a local temperature of battery exceeds 55 °C, a large temperature gradient will form between the battery and its environment, which would effectively help a self‐cooling of battery. In addition, the low‐flammability or even fire‐extinguishing property arising from the use of concentrated electrolytes will undoubtedly reduce any risk of thermal runaway. Therefore, by using a concentrated electrolyte, the need for a battery management system is reduced, increasing the energy density and possibly decreasing the cost of the battery system. Based on our results, we encourage further investigation along these lines. For example, additional optimization of the electrolyte composition or the introduction of functional additives would be expected to produce much better battery performance.

## 4 Experimental Section

### Electrolyte and Electrode Preparation

Battery‐grade LiFSA (Nippon Shokubai Co., Ltd.) and DMC (Kishida Chemical Co., Ltd.) were used without further purification. The LiFSA electrolyte solutions were prepared by dissolving LiFSA in DMC in molar concentrations of 1.0 and 4.0 M, respectively, in an Ar‐filled glove box. The commercial 1.0 M LiPF_6_ in EC/DMC electrolyte (1:1 by volume) was purchased from Kishida Chemical Co., Ltd. and used as a reference.

NMC622 (Hosen Corp.), natural graphite (SEC Carbon), acetylene black (AB, Li‐400, Denka Company Limited), polyvinylidene difluoride (PVdF, Kureha), and *N*‐methylpyrrolidone (NMP, Wako) were purchased and used to prepare the electrodes. The cathode was prepared by mixing NCM622 with AB and PVDF in NMP in an NMC622:AB:PVdF weight ratio of 87:8:5. The anode was fabricated by mixing the natural graphite with PVDF at a weight ratio of 90:10 in NMP. The slurries were coated on an Al foil (20 *μ*m) current collector for the NCM622 cathode and on a Cu foil (10 *μ*m) current collector for the graphite anode using a doctor blade. Both electrodes were dried at 60 °C first for 2 h and, then, further dried at 120 °C under vacuum overnight before use. The active material mass loading was 2–3 mg cm^−2^ for graphite anode in all tests and 2–3 mg cm^−2^ for the NCM622 cathode in all tests except for the full cell tests, for which an active cathode material mass loading of 7–9 mg cm^−2^ was employed.

### Material Characterization

The solidification temperatures of the solution samples were evaluated by a differential scanning calorimeter (Mettler Toledo DSC 3^+^). The samples were sealed in an Al pan in the Ar‐filled glove box. DSC measurements were tested in a temperature range from −80 to +100 °C with a heating rate of 5 °C min^−1^. The TG tests were carried out using an EXSTAR 6000 analyzer (Seiko Instruments) with a heating rate of 5 °C min^−1^ under Ar protection. The electrolytes were sealed in an Al pan in the Ar‐filled glove box, and a pinhole was punched on the Al pan after loading the sample to allow gas escape during the measurements. The ionic conductivity was studied by AC impedance spectroscopy in a symmetrical Pt|electrolyte|Pt cell using a VMP3 potentiostat (BioLogic) with a frequency range of 100 mHz to 100 kHz over a temperature range from −20 to 120 °C. A standard KCl solution was used to calibrate the cell constant. The solution structure was studied by a Raman spectroscopy with an exciting laser of 514 nm (Witec Alpha 300R). The samples were sealed in a Raman accessary of “Variable‐Temperature Sample Stage” with temperature control from −20 to +100 °C.

The concentrations of metal ions in the electrolyte were detected by ICPMS (iCAP RQ, Thermo Fisher). The morphologies of the graphite anode before and after electrochemical measurements were studied using SEM (Hitachi S4800) at 3 kV. The chemical compositions of the graphite anode were analyzed using XPS (PHI5000 VersaProbe II, ULVAC‐PHI) with monochromatic Al‐*K*_*α*_ X‐rays. For both SEM and XPS, all cells were disassembled in an Ar‐filled glove box, from which the electrodes were extracted, immersed into 3 mL DMC, shaken for 30 s (repeated twice), and then dried under vacuum. Thereafter, the samples were transferred from the Ar‐filled glove box to instrumental chambers using an instrumental accessory of “Sample Transfer Vessel” without exposure to air.

### Electrochemical Measurements

Graphite|Li, NCM622|Li half‐cells, and NCM622|graphite full cells were assembled in standard 2032‐type coin cells using an Ar‐filled glove box. Polyethylene separator was used in both the half‐cells and full cells. The volume of the electrolyte was 120 *μ*L, which was sufficient to wet the separators and electrodes fully. In the full cells, the capacity ratio of NCM622: graphite was controlled at 1:1. Galvanostatic charge–discharge cycling and rate capability tests were performed using a TOSCAT‐3100 unit (Toyo System Co.). All cells were kept at the test temperature for 8 h before charge–discharge tests. Charge and discharge measurements were conducted at the same C‐rate without constant voltage mode and at the same temperature. Electrochemical impedance spectroscopy (EIS) was carried out on the graphite|Li half‐cells with an amplitude of 5 mV over a frequency range of 1 MHz to 0.3 mHz. The cells, with 33% state of charge (intercalation), were kept at the test temperature for 5 h to reach equilibrium before EIS measurements.

## Conflict of Interest

The authors declare no conflict of interest.

## Author Contributions

J.W., Q. Z., and M.F. contributed equally to this work. J.W., Y.Y., and A.Y. proposed the concept. J.W. and Q.Z. designed the experiments. J.W., Q.Z., M.F., and S.K. conducted the experiments. All authors contributed to the discussion. J.W., Q.Z., Y.Y., and A.Y. wrote the manuscript. A.Y. supervised the overall project.

## Supporting information

Supporting Information

## Data Availability

The data that support the findings of this study are available from the corresponding author upon reasonable request.
