# Supplementary material for: Concentrated Electrolytes Widen the Operating Temperature Range of Lithium‐Ion Batteries
Source: Adv Sci (Weinh). 2021 Jul 23;8(18):2101646. doi: 10.1002/advs.202101646 (PMC8456280; doi:10.1002/advs.202101646)
Supplement: Supplementary file 1 — Supporting Information [file ADVS-8-2101646-s001.pdf]

© 2021 Wiley-VCH GmbH

ADVANCED SCIENCE
Open Access

Supporting Information

for *Adv. Sci.*, DOI: 10.1002/advs.202101646

Concentrated electrolytes widen the operating temperature range of lithium-ion batteries

*Jianhui Wang, Qifeng Zheng, Mingming Fang, Seongjae Ko, Yuki Yamada, and Atsuo Yamada**

Supporting information

Concentrated electrolytes widen the operating temperature range of lithium-ion batteries

Jianhui Wang[‡]*, Qifeng Zheng*[‡]*, Mingming Fang, Seongjae Ko, Yuki Yamada, and Atsuo Yamada*[*]

Dr. J. Wang, M. Fang

Key Laboratory of 3D Micro/nano Fabrication and Characterization of Zhejiang Province, School of Engineering, Westlake University, Hangzhou, China

Dr. Q. Zheng

School of Chemistry, South China Normal University, Guangzhou, China

Dr. S. Ko, Dr. Y. Yamada, Prof. Yamada

Department of Chemical System Engineering, University of Tokyo, Tokyo, Japan

Elements Strategy Initiative for Catalysts and Batteries, Kyoto University, Kyoto, Japan

Email: yamada@chemsys.t.u-tokyo.ac.jp

[‡]Dr. J. Wang and Dr. Q. Zheng contributed equally to this work.

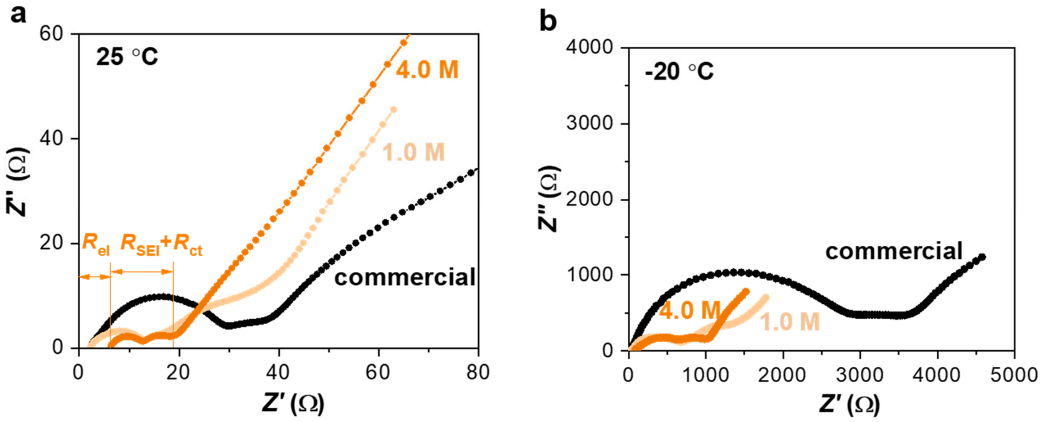

Figure S1. Impedance spectra of the graphite|Li half-cells with various electrolytes at 25 °C (a) and -20 °C (b). The *x*-axis intercept is attributed to the electrolyte bulk resistance (R_{el}). The two semicircles are composed of the solid–electrolyte interphase (SEI) resistance (R_{SEI}) and charge-transfer resistance (R_{ct}). At 25 °C, the cell with 4.0 M LiFSA/dimethyl carbonate (DMC) shows larger R_{el} but much smaller R_{SEI} + R_{ct} than those with 1.0 M LiFSA/DMC and the commercial electrolyte, leading to the lowest total cell resistance. At -20 °C, the total cell resistance is primarily dominated by R_{SEI} and R_{ct} because R_{el} becomes negligible compared to R_{SEI} and R_{ct}.

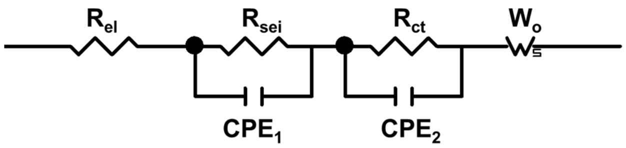

Table S1. The electrolyte bulk resistance (R_{el}), solid–electrolyte interphase resistance (R_{SEI}) and charge-transfer resistance (R_{ct}) obtained from equivalent circuit fitting.

Electrolyte	Temperature (°C)	R_{el} (Ω)	R_{SEI} (Ω)	R_{ct} (Ω)
1.0 M $LiPF_6$/EC:DMC	−20	12.6	3872	2494
	25	3.0	22.8	156.4
1.0 M LiFSA/DMC	−20	30.9	543.5	3544
	25	2.2	9.4	262.1
4.0 M LiFSA/DMC	−20	77.2	431.7	701.8
	25	6.2	4.1	51.0

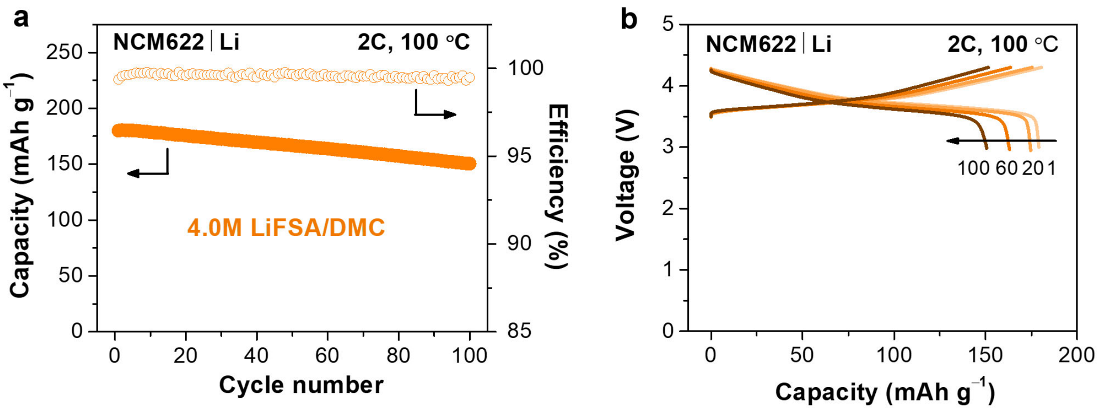

Figure S2. High-temperature operation of a NCM622|Li half-cell. (**a**) Cycling performance of the half-cell with 4.0 M LiFSA/DMC at 2C and 100 °C. **(b)** Selected charge–discharge curves for the half-cell.

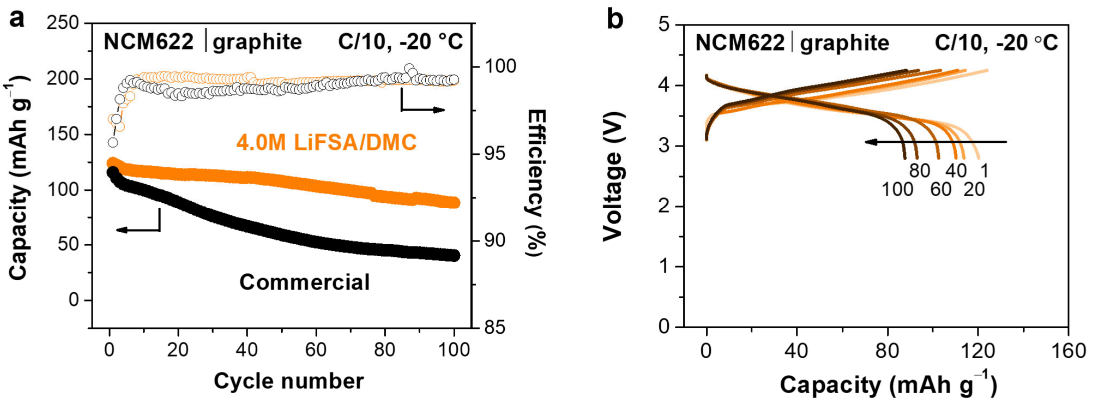

Figure S3. Low-temperature operation of a NCM622|graphite full cell. (**a**) Cycling performance of the full cell with 4.0 M LiFSA/DMC and commercial 1.0 M $LiPF_6$/EC:DMC at C/10 and -20 °C. **(b)** Selected charge–discharge curves for the full cell with 4.0 M LiFSA/DMC at -20 °C.

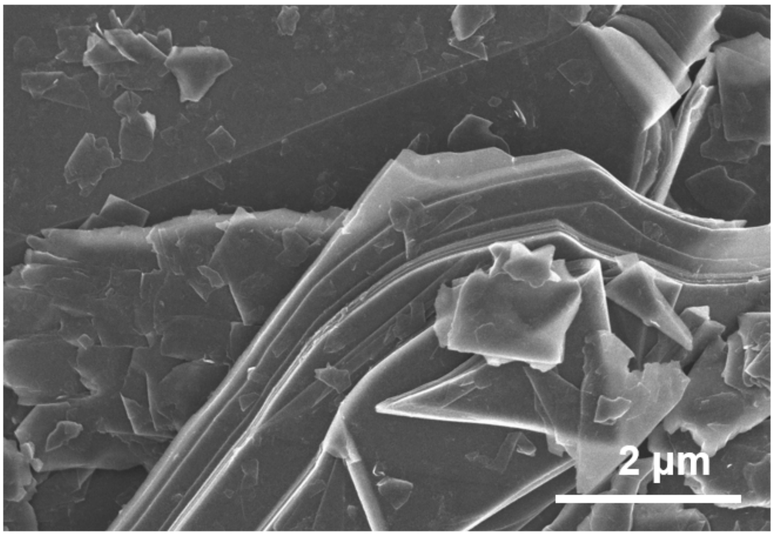

Figure S4. SEM images of the as-prepared graphite electrode.

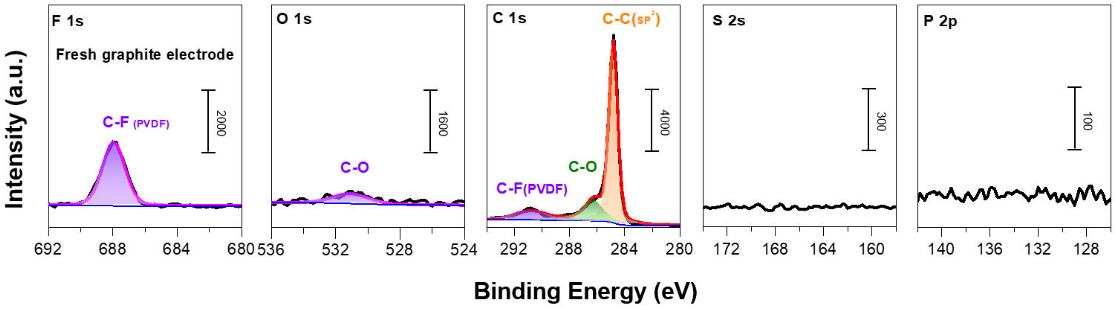

Figure S5. X-ray photoelectron spectra of the as-prepared graphite electrode.

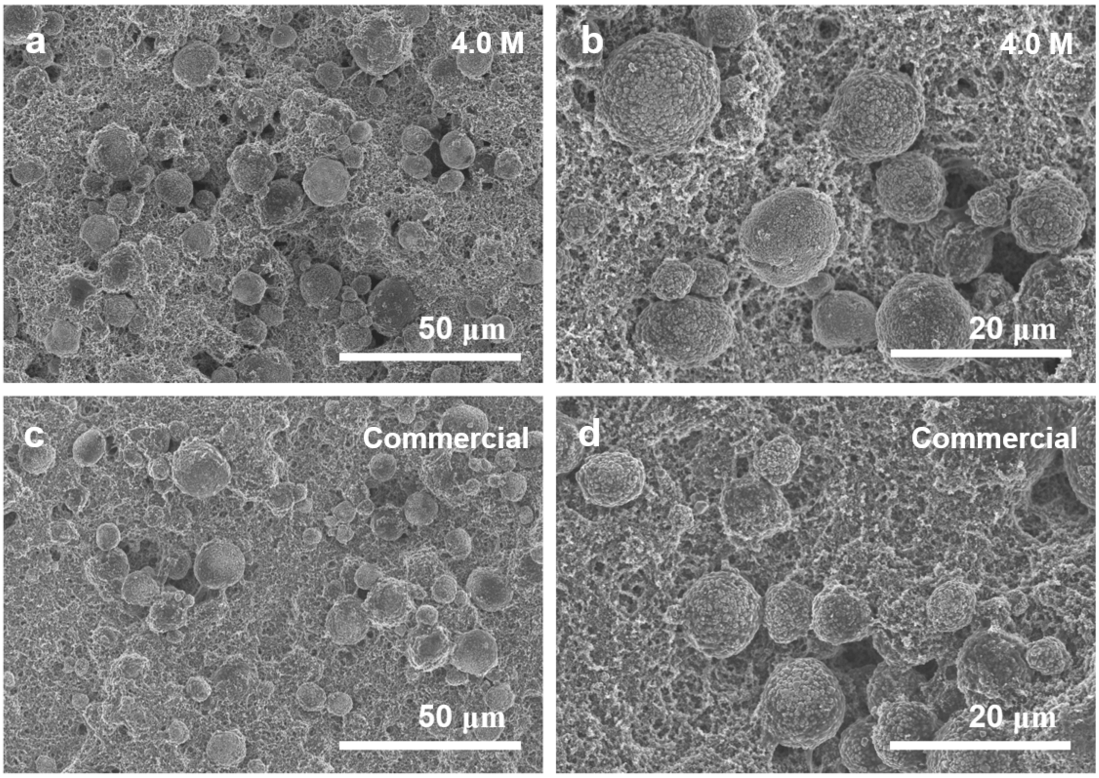

Figure S6. SEM images of the NCM622 electrodes after cycling at 25 °C. (a, b) are SEM images of NCM622 electrodes cycled in the concentrated 4.0 M LiFSA/DMC electrolyte. **(c, d)** are SEM images of NCM622 electrodes cycled in the commercial 1.0 M $LiPF_6$/EC:DMC (1:1) electrolyte.

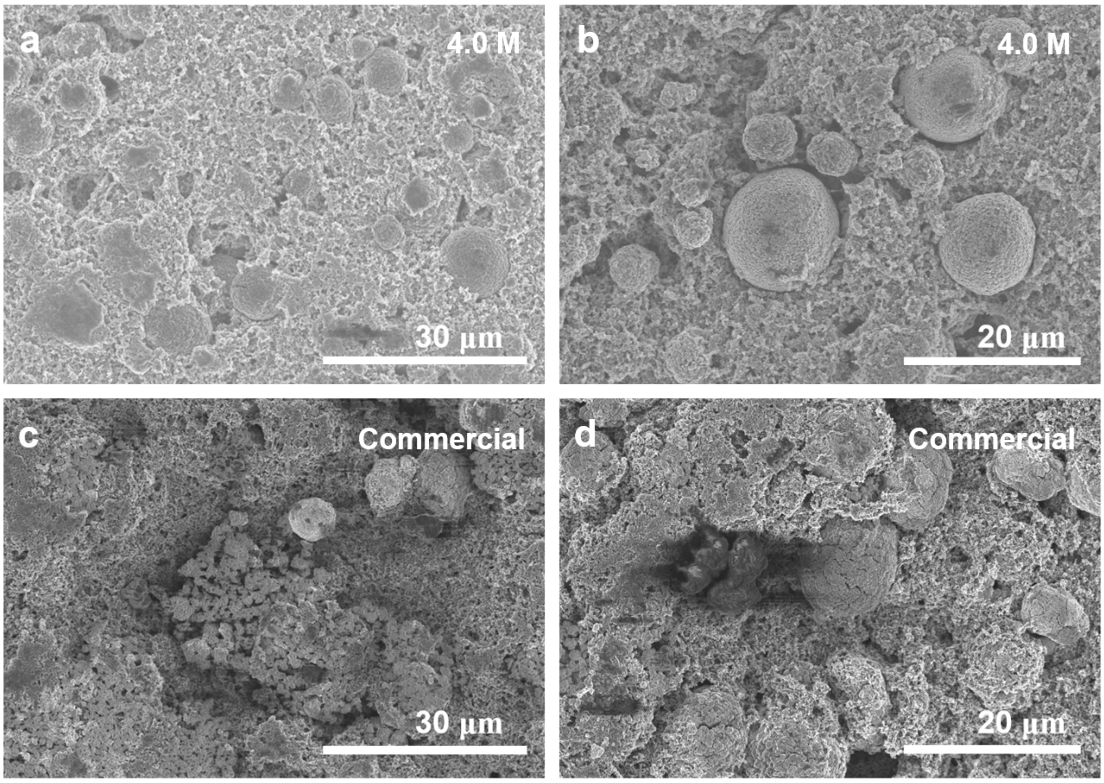

Figure S7. SEM images of the NCM622 electrodes after cycling at 100 °C. (a, b) are SEM images of NCM622 electrodes cycled in the concentrated 4.0 M LiFSA/DMC electrolyte. **(c, d)** are SEM images of NCM622 electrodes cycled in the commercial 1.0 M $LiPF_6$/EC:DMC (1:1) electrolyte.

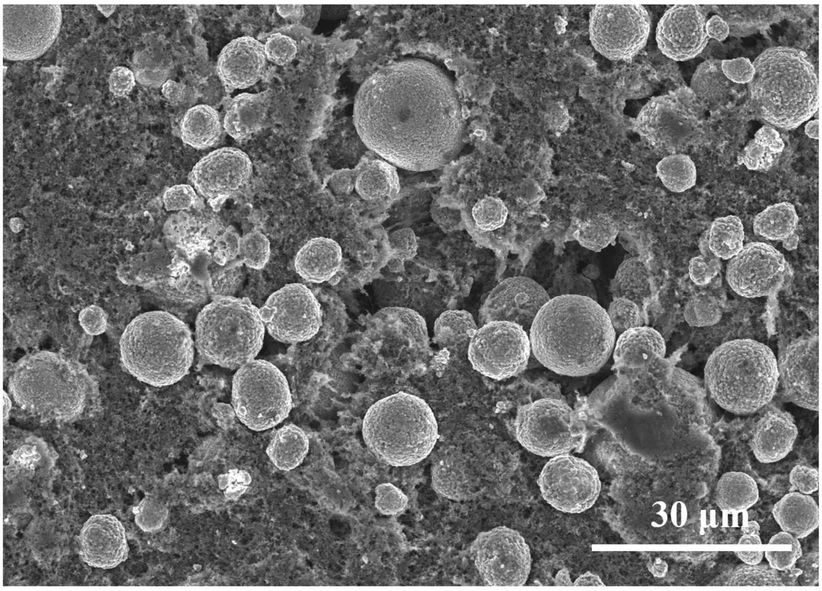

Figure S8. SEM images of the as-prepared NCM622 electrode.
